# Prognostic relevance of autophagy markers LC3B and p62 in esophageal adenocarcinomas

**DOI:** 10.18632/oncotarget.9649

**Published:** 2016-05-26

**Authors:** Olivia Adams, Bastian Dislich, Sabina Berezowska, Anna M. Schläfli, Christian A. Seiler, Dino Kröll, Mario P. Tschan, Rupert Langer

**Affiliations:** ^1^ Institute of Pathology, University of Bern, Bern, Switzerland; ^2^ Graduate School for Cellular and Biomedical Sciences, University of Bern, Bern, Switzerland; ^3^ Department of Visceral Surgery and Medicine, Inselspital University Hospital Bern and University of Bern, Bern, Switzerland

**Keywords:** esophageal adenocarcinoma, autophagy, LC3B, p62, Pathology Section

## Abstract

Esophageal adenocarcinomas (EAC) are aggressive tumors with considerable rates of chemoresistance. Autophagy is a lysosome-dependent degradation process, characterized by the formation of vesicles called autophagosomes, and has been implicated in cancer. Protein light chain 3 B (LC3B) and p62 are associated with autophagosomal membranes and degraded. We aimed to assess the impact of basal autophagy on EAC. In EAC cell lines, an increase in LC3B and p62 was observed with increasing concentrations of the autophagy inhibitor chloroquine, which indicates functional basal autophagy. LC3B and p62 immunohistochemistry was performed on primary resected EAC. High LC3B and p62 expression was associated with earlier tumor stages (*p* < 0.05). High nuclear and cytoplasmic p62 staining were associated with a better prognosis (*p* = 0.006; *p* = 0.028). Various combinations of p62 expression with or without LC3B expression identified different prognostic groups. Tumors with low total p62 (*p* = 0.007) or low LC3B/low p62 expression had the worst outcome (*p* = 0.007; *p* = 0.005). A combination score of dot-like/cytoplasmic p62 and nuclear p62 staining was an independent prognostic parameter (*p* = 0.033; HR = 0.6). This study highlights the potential significance of basal autophagy in EAC biology. Tumors with low LC3B and p62 expression show the most aggressive behavior and may be candidates for autophagy regulating therapeutics.

## INTRODUCTION

Esophageal Adenocarcinomas (EAC), usually arising from metaplastic Barrett's esophagus (BE), are highly aggressive tumors and often locally and systemically advanced upon diagnosis. Furthermore, the incidence is increasing in developed countries [1]. Treatment for locally advanced disease has improved with refined surgical techniques and the advent of neoadjuvant chemotherapy. However, the therapeutic options for recurrent and metastatic disease are mainly limited to (radio)chemotherapy leading to an overall poor prognosis for EAC [2]. Alternative therapeutic strategies, which also may encompass targeting molecular events/regulation, would therefore be highly desirable.

Macroautophagy (hereafter referred to as autophagy) is a cellular catabolic process for the degradation and recycling of cellular components such as proteins and organelles. The process is tightly regulated and highly conserved. Under basal conditions autophagy maintains homeostasis. Autophagy is a pro-survival mechanism under cellular stresses, such as nutrient deprivation, as it frees up building blocks and energy for essential processes [3]. The dysregulation of autophagy has been implicated in many diseases including neurodegenerative diseases. In cancer autophagy has demonstrated diverging roles, being described as both pro- and anti-oncogenic. It is theorized that autophagy can have a tumor suppressor function in early stages as it degrades deleterious proteins and maintains genomic stability. However, later on autophagy can be exploited by neoplastic cells as a survival mechanism enabling progression, invasion, metastasis and evading cell death upon treatment [4]. Autophagy also has diverging roles with respect to tumor immune surveillance given its effect on the tumor microenvironment and function in antigen presentation [5, 6]. Therefore the first aim of this study was to confirm functional basal autophagy in EAC cell lines. Functional basal autophagy can be defined as the continual autophagic flux under steady state culture conditions in the absence of chemo-, radio or targeted therapy. This is of relevance given the recent characterization of cancer cell lines lacking core autophagy machinery components and consequently proving unable to undergo functional basal autophagic flux [7, 8]. While cancer cell lines lacking functional basal autophagy are rare, in light of recent discoveries, this proof of principle experiment was of import. Functional basal autophagy can be assessed *via* the markers LC3B and p62 in the absence and presence of pharmacological autophagy inhibition. Cytosolic LC3B-I is lipidated and incorporated into the autophagosomal membrane, forming the LC3B-II isoform. LC3B-II is subsequently degraded during autophagic flux [9]. As LC3B-II is membrane-bound and subsequently degraded, the accumulation of this isoform upon pharmacological autophagy inhibition is indicative of intact basal autophagy. LC3B-II can also be visualized *via* immunofluorescence as punctae. Similarly, the accumulation of p62, which is also associated with the autophagosomal membrane and subject to degradation, upon pharmacological autophagy inhibition is also an indicator of functional basal autophagy [9]. The second aim was to assess basal autophagy in EAC *ex vivo.* Immunohistochemical LC3B and p62 dot-like staining is considered to be indicative of autophagosomes and may therefore be used as markers in patient tissue. Using a previously established staining and scoring protocol [10] LC3B and p62 immunohistochemistry was performed on next generation tissue microarrays (ngTMAs) of formalin fixed paraffin embedded (FFPE) samples of primary resected EAC, Barrett's mucosa, adjacent non-neoplastic esophageal mucosa, adjacent non-neoplastic gastric mucosa, EAC lymph node metastasis and EAC distant metastasis and the results were then correlated with clinicopathological parameters.

## RESULTS

### EAC exhibits intact basal autophagy *in vitro*

**Figure 1:**
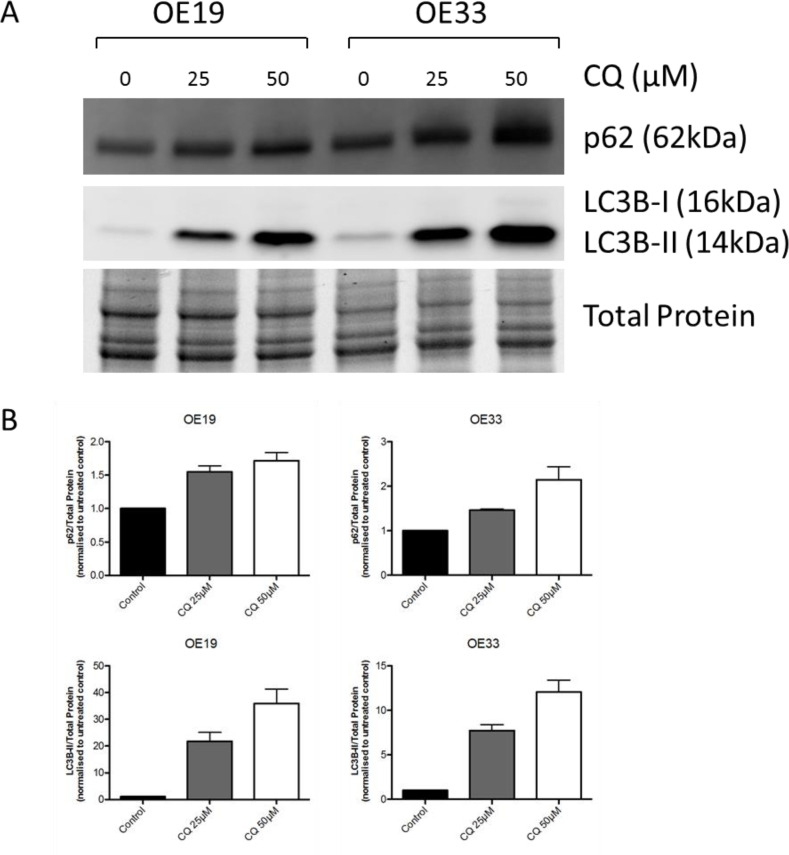
EAC exhibits intact basal autophagy *in vitro* as determined by immunoblotting of markers LC3B and p62 upon pharmacological autophagy inhibition **A.** EAC cell lines OE19 and OE33 were treated with increasing concentrations of the autophagy inhibitor chloroquine (CQ) for 48hr followed by immunoblotting of the autophagy markers p62 and LC3B. Total protein was visualized as loading control. Representative blot is shown. **B.** Quantification of triplicate repetitions of experiments shown in A.

**Figure 2:**
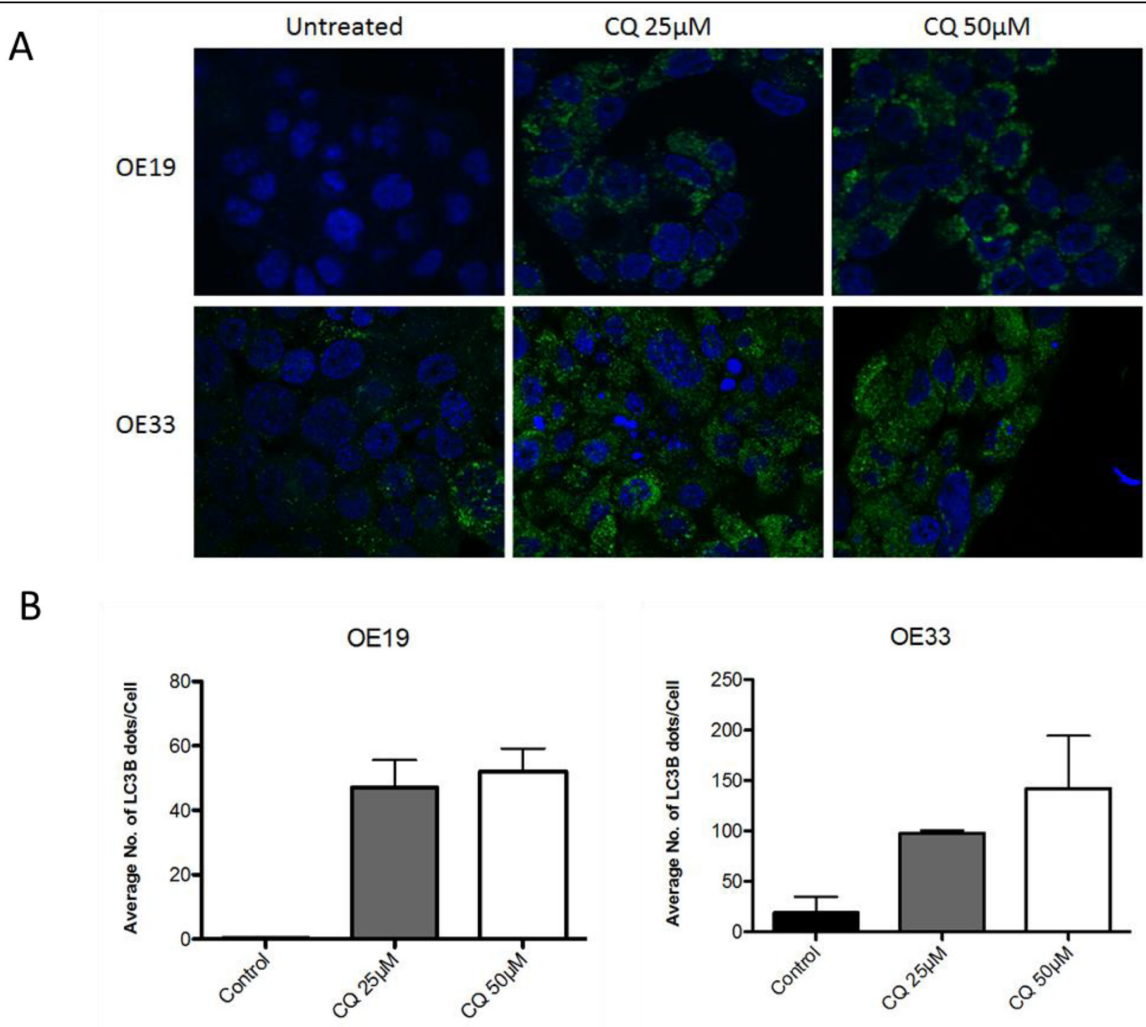
EAC exhibits intact basal autophagy *in vitro* as determined by immunofluorescent staining of LC3B upon pharmacological autophagy inhibition **A.** OE19 and OE33 were treated with increasing concentrations of CQ for 48hr and LC3B detected *via* immunofluorescence. LC3B-II is visualized as green punctae, representing autophagosomes. Nuclei were stained with DAPI (blue). Representative images are shown. **B.** Quantification of triplicate repetitions of experiments shown in A.

To access functional basal autophagy in EAC *in vitro* the autophagy markers LC3B and p62 were assessed *via* immunoblotting and immunofluorescence upon pharmacological autophagy inhibition with chloroquine. EAC cell lines OE19 and OE33 were treated with chloroquine (CQ) for 48 hours followed by immunoblotting for LC3B and p62, with total protein was visualized as loading control. A dose-dependent increase of both proteins was observed with increasing concentrations of CQ, which is indicative of functional basal autophagy (Figure 1). This result was verified by immunofluorescence staining in both cell lines as a dose-dependent increase in LC3B-II punctae with increasing concentrations of CQ was observed, confirming functional basal autophagy (Figure 2).

### Clinicopathological and prognostic implications of autophagy markers in EAC tissue

Median age of the patient cohort was 69 years (ranging from 32 to 89 years). Male/female ratio was 100/16. The pT category (according to UICC 2009 [11]) was as follows: pT1 **-** 35 cases (30.2%), pT2 **-** 11 cases (9.5%), pT3 - 67 cases (57.8%) and pT4 3 cases (2.6%). Lymph node metastases were absent in 56 cases (48.3%) and present in 60 cases (51.7%). Distant metastases at the time of surgery were present in 5 cases (4.3%). Lymphatic invasion was present in 78 cases (67.2%), venous invasion in 29 cases (25%) and perineural invasion present in 45 cases (38.8%). Tumor grading was classified as G1 (well differentiated) in 18 cases (15.4%), G2 (moderately differentiated) in 50 cases (43.1%) and G3 (poorly differentiated) in 48 cases (41.4%). Intratumoral inflammation was low in 55 cases (47.4%) and high in 61 cases (52.6%). Complete tumor resection (R0) was achieved in 109 cases (93.9%). The median overall survival, which was calculated from the day of surgical resection until last contact or death, was 28 months (ranging from 0 to 208 months). Patient data is summarized in Table 1.

**Table 1:** Summary of patient data

Parameter	Category	Total	Percentage (%)
Median age	-	69 years	-
Sex	Male Female	100 16	86.2 13.8
pT category (T)	pT1 pT2 pT3 pT4	35 11 67 3	30.2 9.5 57.8 2.6
Lymph Node Metastasis (N)	pN0 pN1 pN2 pN3	56 19 22 19	48.3 16.4 19.0 16.4
Distant Metastasis (M)	absent present	111 5	95.7 4.3
UICC Stage	IA IB IIA IIB IIIA IIIB IIIC IV	32 3 18 7 16 17 18 5	27.4 2.6 15.4 6.0 13.8 14.7 15.5 4.3
Lymphatic Invasion (L)	absent present	38 78	32.8 67.2
Venous Invasion (V)	absent present	87 29	75.0 25.0
Perineural Invasion (Pn)	absent present	71 45	61.2 38.8
Grading (G)	G1 G2 G3	18 50 48	15.5 43.1 41.4
Inflammation	low high	55 61	47.4 52.6
Resection Status (R)	R0 R1	109 7	94.0 6.0

**Figure 3:**
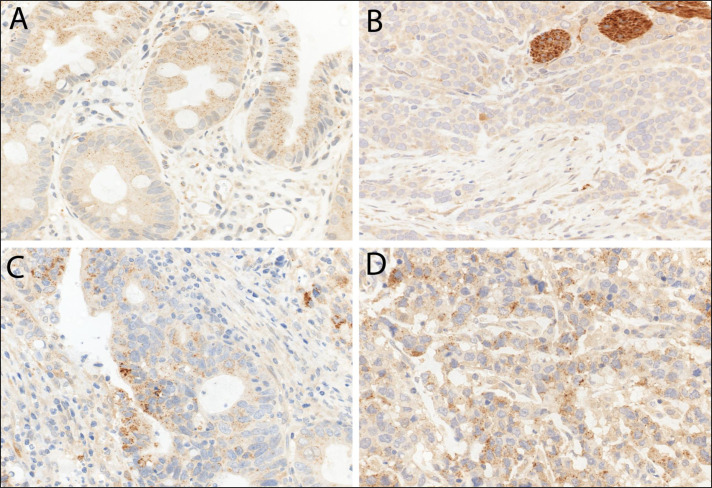
LC3B immunohistochemical staining of Barrett's esophagus (BE) and esophageal adenocarcinoma (EAC) **A.** BE tissue with goblet cells and high LC3B dot-like staining. **B.** EAC tissue with low LC3B dot-like staining and nerves as internal positive control. **C.** and **D.** EAC tissue with high LC3B dot-like staining. Representative images were taken on a Zeiss Axioskop microscope at 40X objective magnification and corrected for brightness.

**Figure 4:**
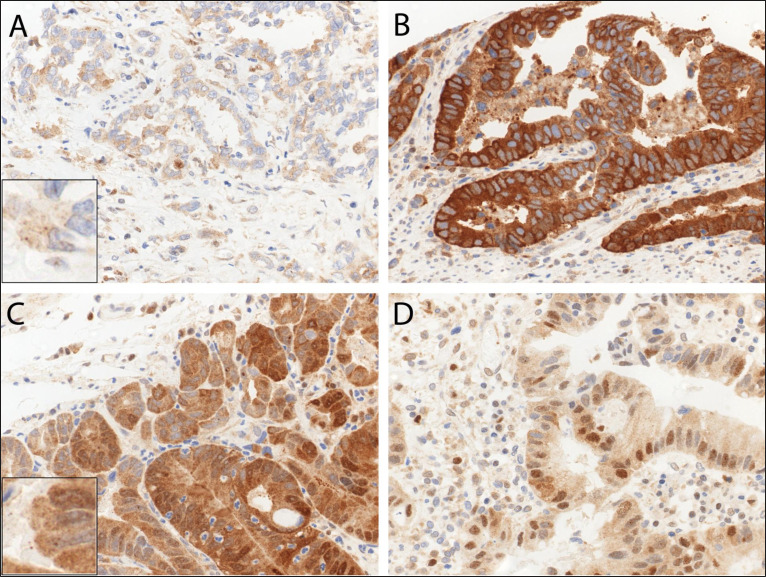
Immunohistochemical staining patterns of p62 in esophageal adenocarcinoma (EAC) EAC tissue exhibiting **A.** low p62 cytoplasmic staining, **B.** high p62 cytoplasmic staining **C.** both high p62 dot-like and diffuse cytoplasmic staining, and **D.** high p62 nuclear positivity. Representative images were taken on a Zeiss Axioskop microscope at 40X objective magnification and corrected for brightness. Insert in **A.** and **C.** is for better visualization of the hardly visible dots.

In primary resected EAC and primary resected non-dysplastic and dysplastic Barrett's mucosa we observed LC3B (Figure 3) and p62 dot-like staining patterns ranging from absent (score 0) to strong (score 3) with detection of abundant immunoreactive dots. Similarly a range of p62 cytoplasmic staining was observed and both negative and positive p62 nuclear staining was reported (Figure 4). LC3B and p62 dot-like immunostaining showed a positive association (*p* = 0.020) in primary resected EAC tumors. A positive association between individual p62 dot-like and p62 cytoplasmic staining scores was observed (*p* = 0.005). See Supplemental material for all correlations between individual staining scores (Supplementary Table 1.1 to Supplementary Table 2.3).

Staining patterns were homogenous for LC3B and all scoring categories of p62 across the tumors. Intratumoral heterogeneity, defined as more than one of the six tumor punches (three taken from tumor center and three taken from tumor periphery) showing discrepancies greater than 1 scoring point, was in the explicit minority. The tumors showed higher amounts of LC3B dots, as well as p62 dot-like, cytoplasmic and nuclear staining, when compared to normal gastric and esophageal mucosa (ranging from *p* < 0.001 to *p* < 0.02). For LC3B there was no significant difference between primary tumor and metastases (*p* = 0.301 for lymph node and *p* = 0.083 for distant metastases). For p62, higher p62 dot-like staining was observed in distant metastases compared to primary tumors (*p* = 0.022) and higher nuclear staining was observed in lymph node metastases compared to primary tumors (*p* = 0.021), whereas no difference was seen between the primary tumors and lymph node and distant metastases for cytoplasmic p62 staining (*p* = 0.835 and *p* = 0.480), neither for p62 dot-like staining regarding lymph node metastases (*p* = 0.777) and for p62 nuclear staining regarding distant metastases (*p* = 0.132).

For the purpose of further analysis IHC scores were categorized as either “low” or “high” based on prognostic impact as outlined in the Materials and Methods section. Categories for low and high for each staining is summarized in Table 2. Low/high groupings of p62 dots and cytoplasmic staining showed a statistically significant correlation (*p* = 0.026). For correlations of all low/high p62 groups please see Supplementary Table 3.1 to 3.3. The association of low and high groups of LC3B dot-like, p62 dot-like, p62 cytoplasmic, p62 nuclear and p62 sum scores with clinicopathological features pT category, presence of lymph node or distant metastases, clinical stage, lymphatic invasion, venous invasion, perineural invasaion, grading, inflammation and resection status was assessed. LC3B dot-like staining did not significantly correlate with any of those clinicopathological features (Table 3). In contrast, low p62 nuclear staining correlated with lymph node metastases (*p* = 0.015), as well as with higher grade (*p* = 0.042; Table 4). Strikingly, no stage I tumors with low p62 cytoplasmic expression were observed (*p* = 0.021; Table 4). In line with this finding, fewer stage I tumors had low p62 nuclear expression (*p* = 0.042; Table 4). Similarly only two stage I tumors had a low p62 sum score (*p* = 0.017; Table 5). Taken together these results indicated that low levels of p62 correlate with a more aggressive phenotype (Table 4; Table 5).

**Table 2:** Summary of staining patterns

Staining Pattern	Low	High
LC3B dots	97 (83.6%)	19 (16.4%)
p62 dots	48 (41.4%)	68 (58.6%)
p62 cytoplasm	12 (10.3%)	104 (89.7%)
p62 nuclear	58 (50.0%)	58 (50.0%)
p62 sum	22 (19.0%)	94 (81.0%)

**Table 3:** LC3B dot-like staining and clinicopathological features

Parameter	Category	Total	LC3B Low	LC3B High	*p* -value
pT category	pT1 pT2 pT3 pT4	35 11 67 3	26 9 59 3	9 2 8 0	0.283
Lymph Node Metastasis	absent present	56 60	47 50	9 10	1.000
Distant Metastasis	absent present	111 5	93 4	18 1	1.000
UICC Stage	I II III IV	35 25 51 5	26 24 43 4	9 1 8 1	0.165
Lymphatic Invasion	absent present	38 78	31 66	7 12	0.790
Venous Invasion	absent present	87 29	73 24	14 5	1.000
Perineural Invasion	absent present	71 45	57 40	14 5	0.305
Grading	G1 G2 G3	18 50 48	12 42 43	6 8 5	0.158
Inflammation	low high	55 61	47 50	8 11	0.802
Resection Status	R0 R1	109 7	90 7	19 0	0.597

**Table 4:** p62 dot-like, p62 cytoplasmic and p62 nuclear staining patterns correlated to clinicopathological features respectively

Parameter	Category	Total	p62 dots			p62 cytoplasm			p62 nuclear		
			Low	High	*p*-value	Low	High	*p*-value	Low	High	*p*-value
pT category	pT1 pT2 pT3 pT4	35 11 67 3	11 6 30 1	24 5 37 2	0.455	0 1 11 0	35 10 56 3	0.070	12 6 38 2	23 5 29 1	0.165
Lymph Node Metastasis	absent present	56 60	23 25	33 35	1.000	5 7	51 53	0.764	21 37	35 23	**0.015**
Distant Metastasis	absent present	111 5	46 2	65 3	1.000	12 0	99 5	1.000	54 4	57 1	0.364
UICC Stage	I II III IV	35 25 51 5	14 11 21 2	21 14 30 3	0.991	0 6 6 0	35 19 45 5	**0.021**	11 13 30 4	24 12 21 1	**0.041**
Lymphatic Invasion	absent present	38 78	18 30	20 48	0.423	4 8	34 70	1.000	18 40	20 38	0.843
Venous Invasion	absent present	87 29	39 9	48 20	0.276	8 4	79 25	0.491	41 17	46 12	0.391
Perineural Invasion	absent present	70 45	29 19	42 26	1.000	7 5	64 40	1.000	31 27	40 18	0.127
Inflammation	low high	55 61	26 22	29 39	0.260	4 8	51 53	0.370	26 32	29 29	0.710
Grading	G1 G2 G3	18 50 48	8 22 18	10 28 30	0.886	0 5 7	18 45 41	0.322	9 18 31	9 32 17	**0.042**
Resection Status	R0 R1	109 7	43 5	66 2	0.124	11 1	98 6	0.544	56 2	53 5	0.438

Statistically significant *p* values are shown in bold.

**Table 5:** p62 sum score and clinicopathological features

Parameter	Category	Total	p62 Sum Low	p62 Sum High	*p* -value
pT category	pT1 pT2 pT3 pT4	35 11 67 3	2 2 18 0	33 9 49 3	0.059
Lymph Node Metastasis	absent present	56 60	9 13	47 47	0.485
Distant Metastasis	absent present	111 5	20 2	91 3	0.239
UICC Stage	I II III IV	35 25 51 5	2 9 9 2	33 16 42 3	**0.017**
Lymphatic Invasion	absent present	38 78	7 15	31 63	1.000
Venous Invasion	absent present	87 29	15 7	72 22	0.421
Perineural Invasion	absent present	71 45	11 11	60 34	0.237
Inflammation	low high	55 61	14 8	41 53	0.102
Grading	G1 G2 G3	18 50 48	3 7 12	15 43 36	0.415
Resection Status	R0 R1	109 7	20 2	89 5	0.616

Statistically significant *p* values are shown in bold.

**Figure 5:**
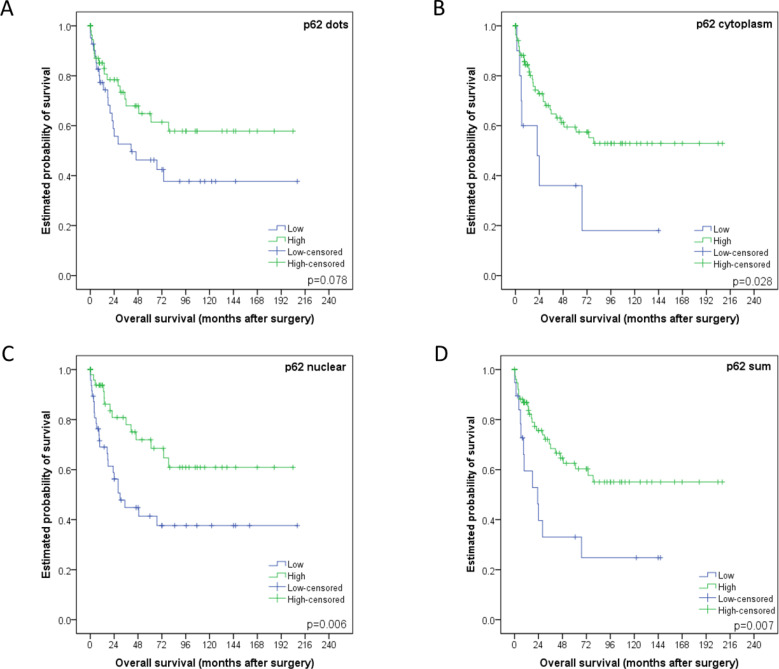
Kaplan-Meier survival curves for p62 staining patterns in esophageal adenocarcinomas **A.** Dot-like, **B.** cytoplasmic or **C.** nuclear staining classified as either low or high. **D.** p62 sum score. For each curve the p-value is displayed on the bottom right-hand corner.

Low/high groupings of staining patterns *versus* overall survival were investigated using univariate analysis. Low p62 cytoplasmic staining, p62 nuclear staining and p62 sum score all significantly correlated with a worse overall survival with *p*-values equaling 0.028, 0.006 and 0.007 respectively (and in trend p62 dot-like staining *p* = 0.078; Figure 5). Low LC3B was associated with worse outcome as well, but this was not statistically significant (*p* = 0.335; see Supplementary Figure 1 for LC3B survival curve).

**Figure 6:**
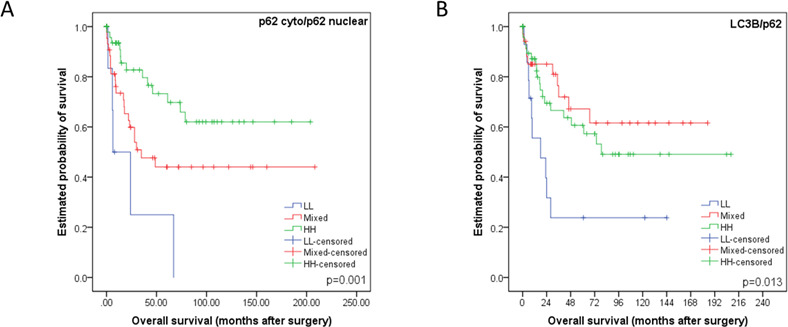
Kaplan-Meier survival curves for subcellular location patterns of autophagy markers p62 and LC3B in esophageal adenocarcinomas **A.** Kaplan-Meier survival curve analysis using classification strategy based on subcellular location and expression of p62. The cohort was subdivided into three groups based on their p62 cytoplasmic (including dot-like) and nuclear staining patterns: low p62 cytoplasmic/low p62 nuclear (LL), either low p62 cytoplasmic/high p62 nuclear or vice versa (mixed), high p62 cytoplasmic/high p62 nuclear (HH). **B.** Kaplan-Meier survival curve analysis using classification strategy based on LC3B dot-like expression and total p62 expression. The cohort was subdivided into three groups: low LC3B/low p62 (LL), either low LC3B/high p62 or vice versa (mixed), high LC3B/high p62 (HH). For each curve the p-value is displayed on the bottom right-hand corner.

According to work published by Iwade *et al.* and Liu *et al.* we subclassified our cohort based on p62 and LC3B expression and subcellular localization of p62 as described in the Materials and Methods section [12, 13]. The p62 cytoplasmic/p62 nuclear LL category correlated with the worst overall survival, the p62 cytoplasmic/p62 nuclear HH category with the best overall survival, while both the HL and LH categories showed intermediate survival rates (*p* = 0.003, data not shown), which justified a combination of these groups into a “mixed” group (Figure 6A). Similarly, the LC3B/p62 LL group faired the worse when compared to HH and mixed subtypes (*p* = 0.013, Figure 6B). When comparing the LL group with the remainder of all other cases grouped together it also showed the worse overall survival (*p* = 0.005, data not shown).

Other prognostic relevant parameters were pT category (*p* = 0.001), pN category (*p* = 0.002), presence of distant metastases (*p* = 0.045), lymphatic vessel invasion (*p* = 0.034), and UICC staging (*p* = 0.001), and in trend the presence of perineural invasion (*p* = 0.058), but not resection status, venous invasion and intratumoral inflammation (*p* = 0.485, *p* = 0.434 and *p* = 0.406 respectively).

In a multivariate analysis encompassing the relevant prognostic factors pT category, lymph node and distant metastases, grading, lymphatic and perineural invasion, the classification strategy of p62 cytoplasmic/p62 nuclear (where cytoplasmic also includes dot-like staining) showed the highest significance level for prognostic discrimination and was an independent prognostic parameter (*p* = 0.033, HR = 0.561; Table 6). p62 cytoplasmic/p62 nuclear staining was also independent prognostic factor in a model encompassing tumor grading and UICC stage (*p* = 0.021; Table 7).

**Table 6:** p62 cytoplasmic/p62 nuclear classification was shown to be an independent prognostic parameter in a multivariate analysis encompassing pT category, pN category, absence or presence of distant metastasis, lymphatic invasion and perineural invasion as well as grading

parameter	HR	95% confidence interval		*p*-value
		min	max	
pT category pT1 *vs* pT2 *vs* pT3 *vs* pT4	1.505	0.916	2.472	0.106
pN category pN0 *vs* pN1 *vs* pN2 *vs* pN3	1.396	0.985	1.977	0.061
distant metastases absent *vs* present	1.684	0.464	6.110	0.428
lymphatic invasion absent *vs* present	1.021	0.374	2.783	0.968
perineural invasion absent *vs* present	0.502	0.211	1.195	0.119
Grading G1 *vs* G2 *vs* G3	1.584	0.935	2.685	0.087
p62 cyto/p62 nucl LL *vs* mixed/HH	0.561	0.329	0.956	**0.033**

Statistically significant *p*-values are shown in bold. HR – Hazard Ratio

**Table 7:** p62 cytoplasmic/p62 nuclear classification was shown to be an independent prognostic parameter in a multivariate analysis encompassing UICC and grading

Parameter	HR	95% confidence interval		*p*-value
		min	max	
UICC stage I *vs* II *vs* III *vs* IV	1.792	1.198	2.682	**0.005**
Grading G1 *vs* G2 *vs* G3	1.451	0.885	2.380	0.140
p62 cyto/p62 nucl LL *vs* mixed/HH	0.549	0.330	0.914	**0.021**

Statistically significant *p*-values are shown in bold. HR – Hazard Ratio

## DISCUSSION

In the present study we aimed to assess the role of basal autophagy in EAC. We observed intact basal autophagy in EAC *in vitro* as we observed a dose-dependent increase of markers LC3B-II and p62 with increasing concentrations of autophagy inhibitor CQ. CQ prevents the fusion of autophagosomes and lysosomes and subsequent degradation. As LC3B-II and p62 is incorporated into the autophagosomal membranes and subsequently degraded, inhibition at a late stage will result in accumulation of these markers and is indicative of functional basal autophagy.

Low p62 staining patterns, regardless of type, subcellular localization or combination with LC3B, correlated with a worse prognosis. Of note, low p62 nuclear staining was associated with lymph node metastases, a higher grade and with higher stage tumors. There was no intratumoral heterogeneity of the staining patterns of our autophagy related markers and no correlation between them and intratumoral inflammatory infiltrates. This was unexpected following the concept that autophagy may be strongly linked to inflammation and may show specific reaction patterns to different environment such as the tumor periphery in contrast to the tumor center [14, 15]. Regarding survival, the classification of our cohort based on p62 cytoplasmic/p62 nuclear staining patterns was an independent prognostic factor with a significantly worse outcome in patients with low cytoplasmic/low nuclear p62 expressing tumors.

At present the literature focusing on the role of autophagy in EAC is limited. O'Donovan *et al.* [16] reported that chemoresistant esophageal cancer cell lines induce cytoprotective autophagy upon treatment. A recent follow up study [17] added that pharmacological autophagy inducers rapamycin and lithium had diverging effects when combined with chemotherapeutic agents in esophageal cancer cell lines. Given the different research settings it is difficult to situate our current work in relation to the aforementioned studies. Roesley *et al.* [18] reported that dysplastic BE and EAC have lower levels of Beclin-1, an early autophagy initiator, when compared to non-dysplastic BE and normal squamous mucosa. This contrasts with our study as we observed significantly lower levels of LC3B and p62 in normal esophagus and gastric mucosa, when compared to the tumor tissue in our cohort. We found the autophagy machinery in EAC under basal conditions to be intact *in vitro*. Preliminary experiments performed in our group confirmed that pharmacological and amino acid starvation autophagy induction in EAC cell lines OE19 and OE33 (data not shown). This is in contrast to previous reports by Roesley *et al*., stating that dysplastic BE and EAC displays resistance to autophagy inducers. These discrepancies can be explained by different experimental set ups being used in previous work that may not adequately discriminate between an induction or a block of autophagy. We observed that lower levels of LC3B and p62 correlated with more advanced staged tumors. In addition we observed higher levels of both markers, when compared to normal matched tissue, in a small sample collection of non-dysplastic and dysplastic BE tissue (data not shown). Although we did not explicitly investigate the role of autophagy in the progression of Barrett's esophagus, these preliminary findings are indicative for an important role of autophagy in EAC pathogenesis and warrant further investigation.

El-Mashed *et al.* identified LC3B cytoplasmic, LC3B ring-like structures and LC3B stone-like structures in treatment naïve and neoadjuvant treated EAC cohorts [19]. LC3B cytoplasmic staining correlated with a better overall survival, which is contrast to our study as low LC3B dot-like structures, in combination with low p62 expression, correlated with a shorter overall survival. We did not observe globular structures, while only observing ring-like structures in a very small subset of cases without prognostic significance. The difference in staining patterns observed could be explained by the different specificity and reactivity of the antibodies used in each study. El-Mashed *et al.* used anti-LC3B antibody from Abgent (rabbit polyclonal, directed at N-terminal 1-30 amino acids, AP1802a), while anti-LC3B antibody from Novus Biologicals (rabbit polyclonal, directed at N-terminal #NB600-1384) was used in our study. It has also been published that the anti-LC3B antibody from Novus Biologicals shows crossreactivity with LC3A [20]. It has been demonstrated that the different isoforms of LC3 is differentially expressed in various tissues, however little is known about the expression patterns in esophageal normal and neoplastic tissue. Furthermore, it has been shown that both LC3A and LC3B localized to autophagosomal membranes upon induction and are therefore suitable markers [21, 22]. While the crossreactivity of the antibody in this study can be considered a confounding factor, the interpretation of staining patterns remains the same as both isoforms are markers for autophagic activity. This highlights the importance of standardization of staining and scoring protocols of LC3B prior to widespread implementation as a routine biomarker.

Our finding that low p62 expression correlates with a more aggressive phenotype, worse prognosis and worse overall survival in EAC is in contrast to numerous studies done on other tumor entities [23–27], although there are also diverging reports about a potential prognostic impact of p62 [13]. At present, p62′s role in cancer has to be considered context dependent and elucidating the underlying mechanism is complicated given the multiple functions of p62.

It is well described that p62 functions as an adaptor protein, which binds ubiquitinated protein aggregates and delivers it to autophagosomes *via* its association with LC3B [28]. In addition, p62 has been identified as a major player in other pathways. This includes the nuclear factor erytheriod-derived-2-like 2 -Kelch-like ECH-associated protein 1-antioxidant response element (Nrf2-Keap1-ARE) pathway, where dysregulated p62 may contribute to oncogenesis [9, 23]. p62 also plays a tumor promoting role *via* the nuclear factor kappa B (NF-κB) pathway and increased inflammation [29–31]. Given that low cytoplasmic p62 correlates with a worse overall survival in our EAC cohort it seems unlikely that these pathways are contributing factors. p62 has also been described as a tumor suppressor as it facilitates autophagic degradation of regulators of the oncogenic Wnt signalling pathway [32]. It is therefore tempting to speculate that this could be an underlying mechanism for the prognostic impact observed in our cohort. Furthermore, p62 has been shown to be a regulatory player in mitosis and cell cycle regulation [33–35], and has also been described to mediate an alternative nuclear proteolytic degradation pathway [36]. One may speculate that the cases with low nuclear p62 expression, that were associated with a worse prognosis suffered from more nuclear instability/increased nuclear protein aggregation, which contributed to disease progression and an aggressive phenotype.

To our knowledge this is the first study to investigate the prognostic relevance of both autophagy markers LC3B and p62 in primary resected EAC. Investigating the underlying mechanism as to why low p62 expression consistently correlated with a worse patient outcome could lead to a better understanding of EAC biology and open up novel avenues for therapeutic intervention. Basal autophagy, as indicated by low LC3B and p62 expression, may give tumors access to a continuous supply of nutrients and building blocks thereby facilitating progression and an aggressive phenotype. Late stage impairment of basal or activated autophagy, mirrored by mixed or high LC3B and p62 expression, would not confer this oncogenic advantage. Given the divergent roles of autophagy described in neoplastic diseases this is a feasible hypothesis to explain our results. We are aware that assessing the dynamic process of autophagy using a static IHC *ex vivo* approach is a limitation. However, taken together the results described in this study highlight the potential role of autophagy, and other p62 related pathways, in EAC biology and warrant further investigation.

## MATERIALS AND METHODS

### Cell lines, culture and treatment conditions

The human EAC cancer cell lines OE19 and OE33 from the Public Health England Culture Collections were obtained *via* Sigma-Aldrich, Buchs, Switzerland. The cell lines were cultured in RPMI-1640 (Sigma-Aldrich, Buchs, Switzerland) supplemented with 10% fetal bovine serum (Sigma-Aldrich, F7524) and 1% penicillin streptomycin. Chloroquine (Sigma-Aldrich, C6628) was dissolved in distilled water and stored at −20°C. Chloroquine (CQ) inhibits late stage autophagy by increasing lysosomal pH thereby preventing the autophagosome-lysosome fusion and subsequent degradation of autophagic flux markers LC3B-II and p62. Lipidated form of LC3B-II migrates faster than the unlipidated LC3B-I on an SDS-PAGE gel and therefore the two isoforms can be separated and visualized using immunoblotting. Cells were seeded in a 6 well dish and allowed to adhere overnight and CQ was added to the experimental wells the following day at final concentrations of 25μM and 50μM and incubated for 48hr prior to harvesting or fixing of cells for subsequent immunoblotting or immunofluorescence respectively.

### Immunoblotting

OE19 and OE33 cells were washed in phosphate buffered saline (PBS) before lysis in urea buffer (8 M urea, 0.5% tritonX) containing protease inhibitor (complete midi, Roche Diagnostics, Rotkreuz, Switzerland). Samples were then sonicated and centrifuged at 13,000 rcf for 30min. Supernatant was subsequently transferred to a fresh tube. Protein concentration was determined using the Bradford protein assay (BioRad, Cressier, Switzerland). Thirty μg of total protein were denatured in selfmade 5X sample buffer with β-mercaptoethanol (Sigma Aldrich, M-7522) at 95°C for 5 min. Samples were loaded on a 4-20% stain-free pre-cast gel (BioRad) and transferred onto a polyvinylidene difluoride membrane using the Trans-Blot^®^ Turbo™ Transfer system (BioRad). Total protein was visualized as loading control using the ChemiDoc™ MP system (BioRad). Primary antibodies were purchased as follows: the anti-LC3B antibody from Novus Biologicals (rabbit polyclonal, #NB600-1384, LuBioScience, Luzern, Switzerland) and the anti-p62/SQSTM1 antibody from Sigma (mouse monoclonal, clone 2C11, #WH0008878M1, Leiden, The Netherlands). For p62 membranes were blocked in 5% milk/tris-buffered saline (TBS) for 1hr at room temperature (RT), while membranes for LC3B were blocked in 5% bovine serum albumin (BSA)/TBS for 1hr at RT. Working solutions of the primary LC3B and p62 antibodies were prepared with final dilution 1:1000 in 5% milk/TBS with 0.1% Tween (Sigma Aldrich, P9416). Membranes were incubated overnight at 4°C with shaking. DyLight^®^650 conjugated goat anti-rabbit or DyLight^®^550 conjugated goat anti-mouse (LabForce, Muttenz, Switzerland) was used as secondary antibodies and diluted 1:1000 in 5% milk/TBS-T. Membranes were incubated for 1hr at RT with shaking. Proteins of interest were acquired and visualized using the ChemiDoc™ MP system (BioRad). Bands were quantified using ImageJ software (1.64r; NIH, Bethesda, MD, USA).

### Immunofluorescence

OE19 and OE33 was seeded directly onto cover-slides (18mmX18mm) in a 6well plate and allowed to adhere overnight. Cells remained untreated or treated with 25μM and 50μM Chloroquine (CQ) for 48hr prior to fixation. Cells were washed in PBS, pre-fixed in 4% paraformaldehyde for 15 seconds, fixed and permeabilized in ice-cold 100% methanol for 10min at −20°C. Slides were air dried and washed with PBS prior to incubation with the primary anti-LC3B antibody (Cell Signaling, Lausen, Switzerland, rabbit monoclonal, clone D11, #3686,) at a working dilution of 1:100 in PBS/1%BSA/0.1%Tween for 1hr at RT. Subsequently slides were washed twice with PBS 0.1% Tween and once with PBS and incubated for 1hr at RT with the fluorescein isothiocyanate (FITC) conjugated antirabbit secondary antibody (#111-096-045 Jackson Immunoresearch, Suffolk, UK) in a working dilution of 1:130 in PBS/1%BSA/0.1% Tween. Slides were once again washed twice with PBS 0.1% Tween and once with PBS. Slides were allowed to air dry. Mounting media (Thermofisher Scientific, Reinach, Switzerland, S36938) containing DAPI (4′,6-diamidino-2-phenylindole) was added to slides in order to visualize nuclei. Images were taken on a confocal microscope Olympus FluoView1000 at 63x objective magnification. Images were adjusted for brightness and the number of LC3B positive dots was quantified using ImageJ software (1.64r; NIH, Bethesda, MD, USA), using a modified plugin as previously described [37].

### Patients and tissue samples

Patient tissue of esophageal adenocarcinoma resection specimens was collected from the archive of the Institute of Pathology, University of Bern, Bern, Switzerland. The patients were consecutively treated between 1991 and 2011 at the Department of Visceral Surgery of the Inselspital, Bern, Switzerland. We selected only cases that were primarily resected without prior neoadjuvant chemo- or radiochemotherapy or adjuvant treatment. The use of human archival pathological tissue for TMA based studies was approved by the local ethics committee (Kantonale Ethikkommission Bern, Switzerland, 200/14: Identification of novel prognostic and predictive biomarkers using tissue microarrays (TMAs) constructed at the INSTITUTE of Pathology, University of Bern).

### Tissue microarray

Buffered formalin fixed paraffin embedded (FFPE) tissue from primary resected EAC (*n* = 116; three punches taken from tumor center and tumor periphery respectively), Barrett's mucosa (*n* = 21), adjacent non-neoplastic esophageal mucosa (*n* = 60), adjacent non-neoplastic gastric mucosa (*n* = 68), EAC lymph node metastasis (*n* = 58) and EAC distant metastasis (*n* = 18) was used to construct a next generation tissue microarray (ngTMA), with digital annotation of scanned slides and automatic transferal of the punches, as previously described [38]. Three punches from each location were transferred to the TMA acceptor block from each tissue (0.6 mm punches).

### Immunohistochemical staining, scoring and subclassification

The ngTMA was sectioned at 4 μm. After de-paraffination, rehydration, and antigen retrieval, immunohistochemical staining was performed using an automated immunostainer (Bond RX, Leica Biosystems, Heerbrugg, Switzerland) as previously described [10]. Briefly the anti-LC3B antibody (Novus Biologicals #NB600-1384) was diluted 1:4000 in tris buffer and incubated at 95°C for 30 min. The anti-p62/SQSTM1 antibody (MBL rabbit polyclonal, #PM045, LabForce, Nunningen, Switzerland) was diluted 1:9000 in tris buffer and incubated at 95°C for 30 min. Visualization was performed using the Bond Polymer Refine Detection kit (Leica Biosystems, product number) per manufacturer's instructions. IHC stainings were scored according to a previously established protocol [10]. The immunohistochemical stainings were independently scored by two observers (OA and RL) on a Zeiss Axioskop microscope at 40x objective magnification. Discrepant results were re-evaluated at a double-header microscope.

For the purpose of further analysis IHC scores were categorized as either “low” or “high” for each staining pattern according to the prognostic impact of the single scores (for survival curves of individual scores see Supplementary Figures 2 and 3). For LC3B dot-like staining scores 0 and 1 were classified as low, while scores 2 and 3 were classified as high. The low category of p62 dot-like staining was assigned to score 0, while scores 1, 2 and 3 were assigned to the high category. The scores for p62 cytoplasmic staining were similarly subdivided, while score 0 for p62 nuclear staining was classified as low and scores 1 and 2 as high. Finally a p62 sum score was calculated by adding the individual scores of the dot-like, cytoplasmic and nuclear staining patterns together. The p62 sum score ranged from 0 to 6. Subsequently the p62 sum score was also subdivided in low (score 0 and 1) and high (from score 2 to 6) groups.

According to work published by Iwade *et al.* [12] we stratified our dataset into four subtypes with respect to p62 staining patterns and subcellular location: low p62 cytoplasmic/low p62 nuclear (LL), high p62 cytoplasmic/low p62 nuclear (HL), low p62 cytoplasmic/high p62 nuclear (LH) and high p62 cytoplasmic/high p62 nuclear (HH). The cytoplasmic score was calculated by adding the dot-like and cytoplasmic staining scores together with scores 0 and 1 being classified as low and scores 2 through 5 being classified as high (see Supplementary Figure 3). The HL and LH categories were combination into a “mixed” group and used for subsequent analysis. According to Liu and colleagues [13] we stratified our data set according to LC3B and p62 staining patterns, resulting in three groups: low LC3B/low p62 (LL), mixed (low LC3B/high p62 or vice versa) and high LC3B/high p62. In the context of this analysis the p62 sum score low/high categories were used as previously mentioned.

Host inflammatory response to the tumors were scored according to Brown *et al*., who recognized three different grades; i.e. sparse, moderate and pronounced based on histomorphology by Hematoxylin & Eosin staining [39]. For the purpose of this study this grading was simplified into low (sparse) and high (moderate and high) with an equal distribution of the two categories and estimated across all six TMA punches. Examples of low and high inflammatory response are given in Supplementary Figure 5.

### Statistical analysis

The SPSS 23 software (SPSS Inc, Chicago, IL, USA) was used for descriptive and comparative statistical analysis. Associations between staining patterns and clinicopathological parameters were evaluated using simple cross tabs (χ²-test or Fisher's exact test). Binded samples were evaluated using the Wilcoxon test. Survival analysis was performed using log rank test and Cox regression analysis (inclusion). The significance level was set at 0.05.

## SUPPLEMENTARY MATERIAL


