# Supplementary material for: Prognostic relevance of autophagy markers LC3B and p62 in esophageal adenocarcinomas
Source: Oncotarget. 2016 May 26;7(26):39241–55. doi: 10.18632/oncotarget.9649 (PMC5129929; doi:10.18632/oncotarget.9649)
Supplement: Supplementary file 1 [file oncotarget-07-39241-s001.pdf]

Prognostic relevance of autophagy markers LC3B and p62 in esophageal adenocarcinomas

Supplementary Material

Supplementary Table 1.1: Cross-tabulation for LC3B dots and p62 dots individual scores with p-value (bold = significant)

Staining pattern		p62 dots			Total
	**Score**	**0**	**1**	**2**	
**LC3B dots**	**0**	28	23	7	58
	**1**	14	20	5	39
	**2**	6	6	5	17
	**3**	0	0	2	2
**Total**		48	49	19	116
					**p= 0.020**

Supplementary Table 1.2: Cross-tabulation for LC3B dots and p62 cytoplasm individual scores with p-value

Staining pattern		p62 cytoplasm				Total
	**Score**	**0**	**1**	**2**	**3**	
**LC3B dots**	**0**	28	23	12	0	58
	**1**	14	20	11	0	39
	**2**	6	6	4	1	17
	**3**	0	0	0	0	2
**Total**		12	76	27	1	116
						p= 0.167

Supplementary Table 1.3: Cross-tabulation for LC3B dots and p62 nuclear individual scores with p-value

Staining pattern		p62 nuclear			Total
	**Score**	**0**	**1**	**2**	
**LC3B dots**	**0**	33	19	6	58
	**1**	15	17	7	39
	**2**	8	6	3	17
	**3**	2	0	0	2
**Total**		58	42	16	116
					p= 0.473

Supplementary Table 2.1: Cross-tabulation for individual p62 dots and p62 cytoplasmic scores with p-value (bold = significant)

**Staining pattern**		**p62 cytoplasm**				**Total**
	**Score**	**0**	**1**	**2**	**3**	
**p62 dots**	**0**	9	34	5	0	48
	**1**	2	33	14	0	49
	**2**	1	9	8	1	19
**Total**		12	76	27	1	116
						**p= 0.005**

Supplementary Table 2.2: Cross-tabulation for individual p62 nuclear and p62 dots scores with p-value

**Staining pattern**		**p62 nuclear**			**Total**
	**Score**	**0**	**1**	**2**	
**p62 dots**	**0**	23	15	10	48
	**1**	24	20	5	49
	**2**	11	7	1	19
**Total**		58	42	16	116
					p= 0.394

Supplementary Table 2.3: Cross-tabulation for individual p62 cytoplasm and p62 nuclear scores with p-value

**Staining pattern**		**p62 nuclear**			**Total**
	**Score**	**0**	**1**	**2**	
**p62 cytoplasm**	**0**	9	2	1	12
	**1**	32	34	10	76
	**2**	17	5	5	27
	**3**	0	1	0	1
**Total**		58	42	16	116
					p= 0.090

Supplementary Table 3.1: Cross-tabulation for p62 cytoplasm and p62 dots expression levels with p-value (bold = significant)

Staining pattern		p62 dots		Total
	**Expression**	**Low**	**High**	
**p62 cytoplasm**	**Low**	23	35	58
	**High**	25	33	58
**Total**		48	68	116
				**p= 0.026**

Supplementary Table 3.2: Cross-tabulation for p62 nuclear and p62 dots expression levels with p-value

Staining pattern		p62 dots		Total
	**Expression**	**Low**	**High**	
**p62 nuclear**	**Low**	23	35	58
	**High**	25	33	58
**Total**		48	68	116
				p= 0.851

Supplementary Table 3.3: Cross-tabulation for p62 nuclear and p62 cytoplasm expression levels with p-value

Staining pattern		p62 cytoplasm		Total
	**Expression**	**Low**	**High**	
**p62 nuclear**	**Low**	9	49	58
	**High**	3	55	58
**Total**		12	104	116
				p= 0.125

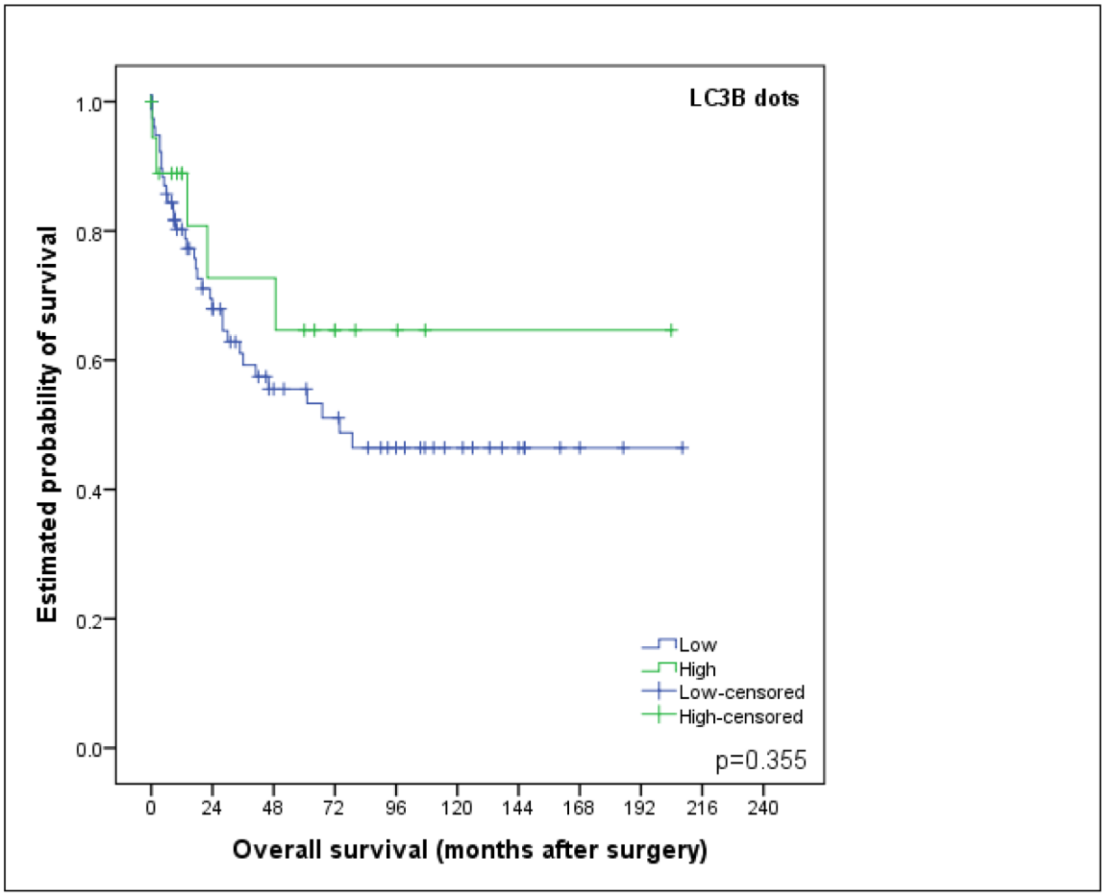

Supplementary Figure 1: Kaplan-Meier survival curves for LC3B dot-like staining patterns (low/high groups) in esophageal adenocarcinomas

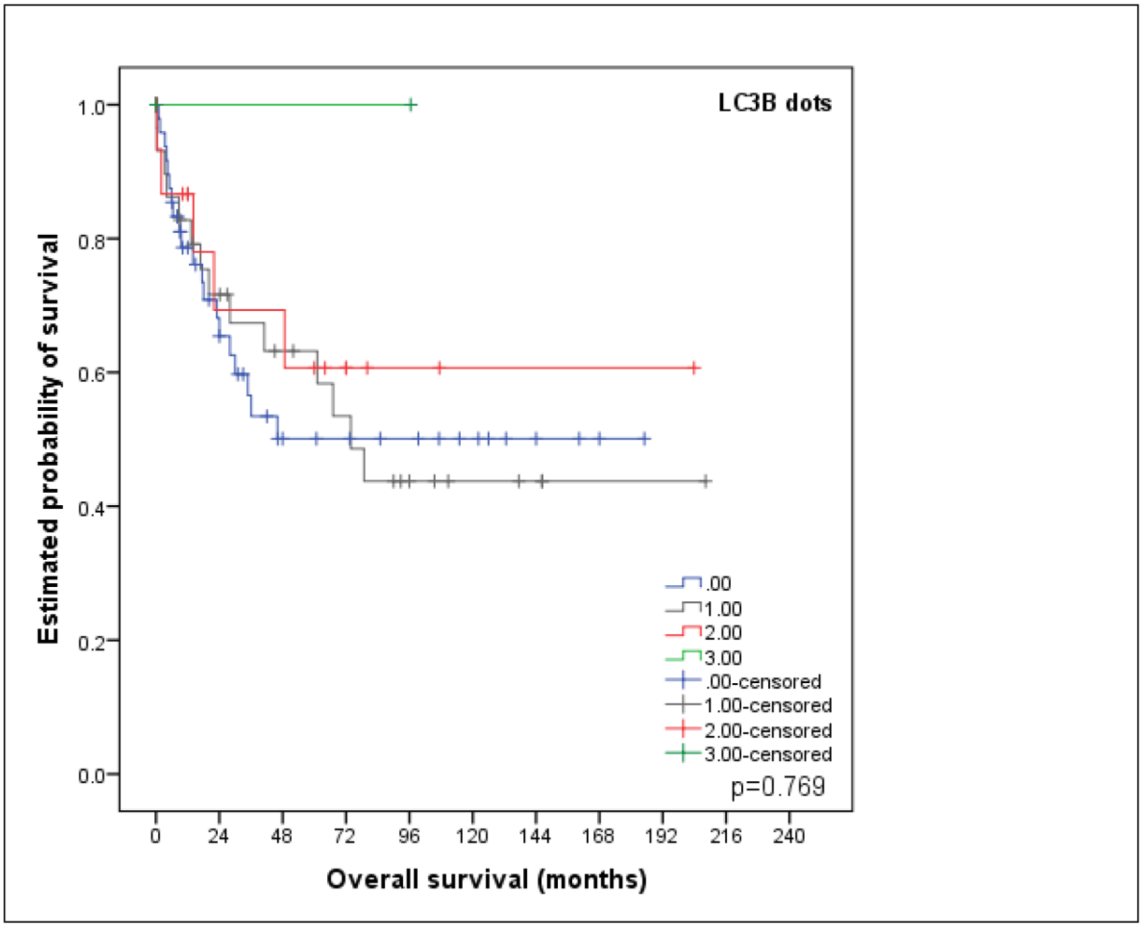

Supplementary Figure 2: Kaplan-Meier survival curves for LC3B dot-like staining patterns (individual scores) in esophageal adenocarcinomas

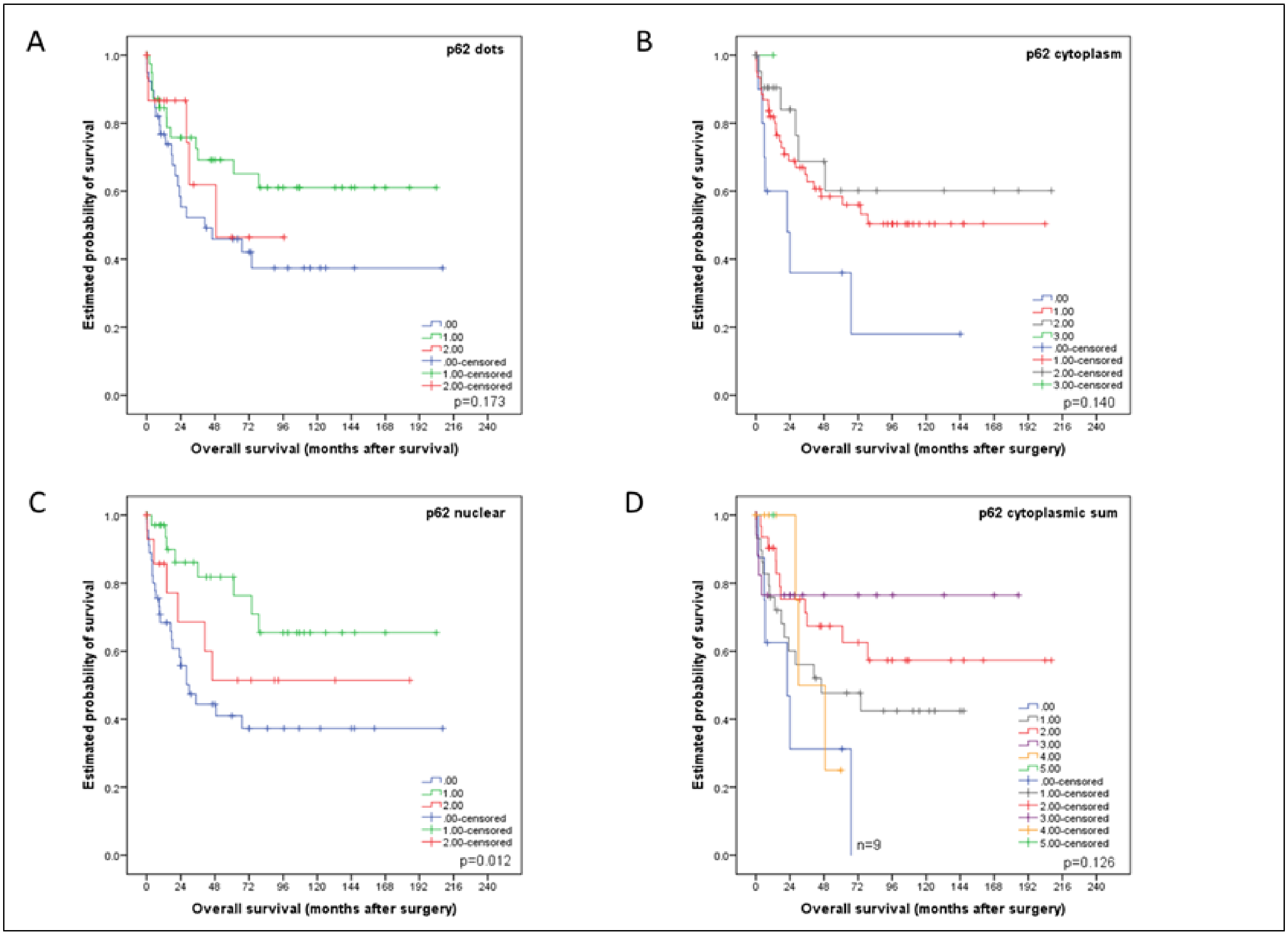

Supplementary Figure 3: Kaplan-Meier survival curves for individual scores of A) p62 dot-like staining, B) p62 cytoplasmic staining, C) p62 nuclear staining and D) p62 cytoplasmic sum score.

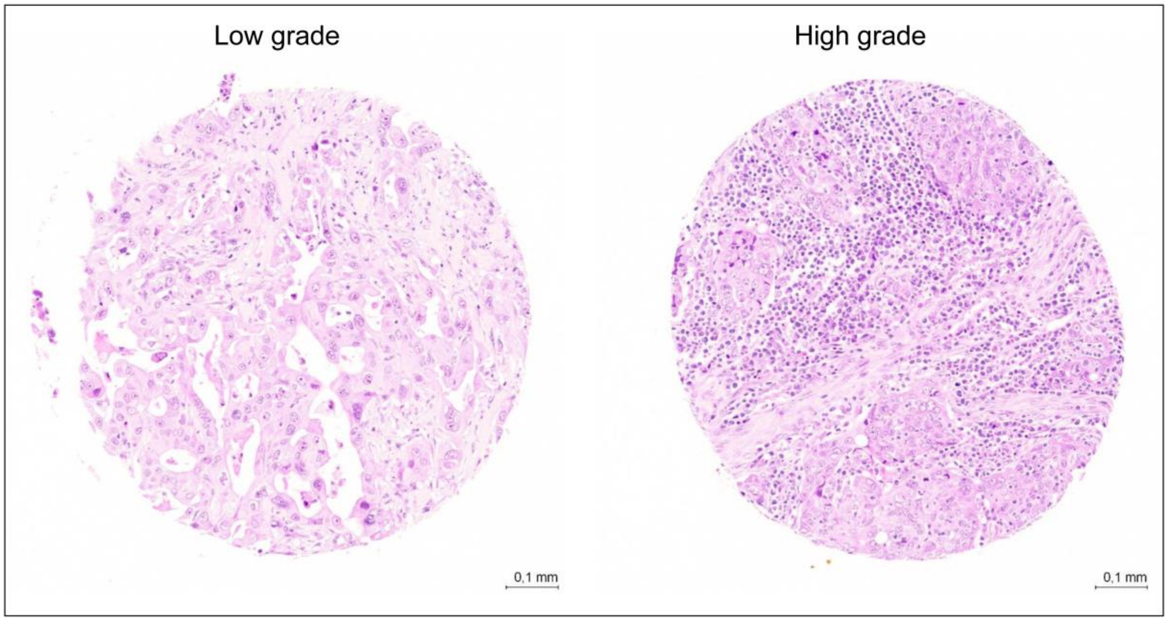

Supplementary Figure 4: Examples of extent of immunoinfiltrates in esophageal adenocarcinomas
